# Improving water, sanitation and hygiene in health-care facilities, Liberia

**DOI:** 10.2471/BLT.16.175802

**Published:** 2017-04-25

**Authors:** Nana Mensah Abrampah, Maggie Montgomery, April Baller, Francis Ndivo, Alex Gasasira, Catherine Cooper, Ruben Frescas, Bruce Gordon, Shamsuzzoha Babar Syed

**Affiliations:** a Service Delivery and Safety Department, World Health Organization, 20 Avenue Appia, 1211 Geneva 27, Switzerland.; b Water, Sanitation, Hygiene and Health Department, World Health Organization, Geneva, Switzerland.; c World Health Organization, Monrovia, Liberia.; d Ministry of Health and Social Welfare, Monrovia, Liberia.

## Abstract

**Problem:**

The lack of proper water and sanitation infrastructures and poor hygiene practices in health-care facilities reduces facilities’ preparedness and response to disease outbreaks and decreases the communities’ trust in the health services provided.

**Approach:**

To improve water and sanitation infrastructures and hygiene practices, the Liberian health ministry held multistakeholder meetings to develop a national water, sanitation and hygiene and environmental health package. A national train-the-trainer course was held for county environmental health technicians, which included infection prevention and control focal persons; the focal persons acted as change agents.

**Local setting:**

In Liberia, only 45% of 701 surveyed health-care facilities had an improved water source in 2015, and only 27% of these health-care facilities had proper disposal for infectious waste.

**Relevant changes:**

Local ownership, through engagement of local health workers, was introduced to ensure development and refinement of the package. In-county collaborations between health-care facilities, along with multisectoral collaboration, informed national level direction, which led to increased focus on water and sanitation infrastructures and uptake of hygiene practices to improve the overall quality of service delivery.

**Lessons learnt:**

National level leadership was important to identify a vision and create an enabling environment for changing the perception of water, sanitation and hygiene in health-care provision. The involvement of health workers was central to address basic infrastructure and hygiene practices in health-care facilities and they also worked as stimulators for sustainable change. Further, developing a long-term implementation plan for national level initiatives is important to ensure sustainability.

## Résumé

**Problème:**

L'absence d'infrastructures d'approvisionnement en eau et d'assainissement adaptées et des pratiques d'hygiène déficientes dans les centres de santé font obstacle à la préparation et à la réponse des centres de santé face à une flambée de maladie et limitent la confiance des communautés envers les services de santé.

**Approche:**

Pour améliorer les infrastructures d'approvisionnement en eau et d'assainissement ainsi que les pratiques d'hygiène, le ministère de la Santé libérien a organisé des réunions avec les différentes parties prenantes afin de créer une série d'interventions nationales dans ces domaines. Une « formation des formateurs » nationale a été dispensée aux techniciens chargés de l'hygiène environnementale dans les comtés, notamment aux référents désignés pour superviser la prévention et le contrôle des risques infectieux ; ces référents ont œuvré en tant qu'acteurs du changement.

**Environnement local:**

Au Libéria, en 2015, seuls 45% des 701 centres de santé étudiés avaient amélioré leur approvisionnement en eau et seulement 27% avaient un système d'élimination des déchets infectieux approprié.

**Changements significatifs:**

La responsabilisation des acteurs locaux -par l'implication des agents de santé locaux- a permis d'assurer le développement et l'affinement de cette série d'interventions. Des collaborations à l'échelle des comtés entre les centres de santé, ainsi que des coopérations multisectorielles, ont permis d'orienter les efforts nationaux, qui se sont traduits par une attention accrue portée aux infrastructures d'eau et d'assainissement et à l'assimilation des bonnes pratiques d'hygiène, dans l’optique d'améliorer la qualité générale des services fournis.

**Leçons tirées:**

Le leadership national a été essentiel pour définir une vision et créer un environnement propice au changement de perception sur l'importance de l'eau, de l'assainissement et de l'hygiène dans la prestation des soins de santé. L'implication des agents de santé a été déterminante pour améliorer les infrastructures et les pratiques d'hygiène de base dans les centres de santé; ils ont également joué un rôle de catalyseurs d'un changement durable. Par ailleurs, afin d'obtenir des améliorations durables, il est important de créer un plan de mise en œuvre sur le long terme pour les initiatives menées à l'échelle nationale.

## Resumen

**Situación:**

La ausencia de infraestructuras adecuadas de agua y saneamiento y unas prácticas higiénicas pobres en centros sanitarios reducen la preparación y respuesta de los centros a brotes de enfermedades y reducen la confianza de las comunidades en los servicios sanitarios ofrecidos.

**Enfoque:**

Para mejorar las infraestructuras de agua y saneamiento y las prácticas higiénicas, el Ministerio de Salud de Liberia celebró reuniones de varias partes interesadas para desarrollar un paquete sanitario nacional de agua, saneamiento e higiene y medioambiente. Se realizó un curso nacional de formación de formadores para los técnicos de salud medioambiental de la región, entre los que se encontraban personas dedicadas a la prevención y control de infecciones; personas que actuaban como agentes de cambio.

**Marco regional:**

En Liberia, únicamente el 45% de los 701 centros sanitarios analizados contaban con una fuente de agua mejorada en 2015, y solo el 27% de dichos centros tenía un servicio adecuado de eliminación de residuos infecciosos.

**Cambios importantes:**

Se introdujo la propiedad local, a través del compromiso de los trabajadores sanitarios de la región, para garantizar el desarrollo y la mejora del paquete. Las colaboraciones regionales entre los centros sanitarios junto con una colaboración de varios sectores a nivel nacional dieron lugar a un mayor enfoque para las infraestructuras de agua y saneamiento y la aceptación de las prácticas higiénicas para mejorar la calidad general de los servicios.

**Lecciones aprendidas:**

El liderazgo a nivel nacional fue importante para identificar una visión y crear un entorno permisivo para cambiar la percepción del agua, el saneamiento y la higiene en los servicios sanitarios. La implicación de los trabajadores sanitarios fue fundamental para abordar las prácticas básicas de infraestructura e higiene en centros sanitarios y también fue un estimulante para lograr un cambio sostenible. Además, el desarrollo de un plan de implementación a largo plazo para iniciativas a nivel nacional es importante para garantizar la sostenibilidad.

## ملخص

**المشكلة:**

يقلل نقص البنية التحتية المناسبة لتوفير المياه والصرف الصحي وغياب ممارسات النظافة والعادات الصحية في مرافق توفير الرعاية الصحية من درجة استعداد المرافق الصحية واستجابتها لتفشي الأمراض، كما يؤدي إلى زعزعة ثقة المجتمعات في الخدمات الصحية المقدمة.

**الأسلوب:**

حرصًا على تحسين البنية التحتية لتوفير المياه والصرف الصحي وممارسات النظافة والعادات الصحية، فقد عقدت وزارة الصحة الليبيرية اجتماعات مع العديد من الجهات المعنية لإعداد حزمة وطنية من خدمات توفير المياه والصرف الصحي والنظافة والعادات الصحية والصحة البيئية. وتم عقد دورة وطنية لتدريب المدربين لفنيي الصحة البيئية في المقاطعة والتي اشتملت على العناصر المهمة والقائمة على تجنب حدوث العدوى ومكافحتها فضلاً عن العناصر المهمة من الأشخاص الذين لعبوا دورًا كبيرًا في إحداث التغيير.

**المواقع المحلية:**

حصلت مرافق توفير الرعاية الصحية في ليبيريا بنسبة تبلغ 45‏% فقط من أصل 701 من المرافق المشاركة في المسح على مصدر مياه محسّن في عام 2015، وحصلت مرافق توفير الرعاية الصحية هذه بنسبة 27‏% على نظام ملائم للتخلص من النفايات لإزالة النفايات المسببة لحدوث العدوى.

**التغيّرات ذات الصلة:**

تم طرح نظام للملكية المحلية من خلال إشراك مجموعة من العاملين المحليين في مجال الصحة لضمان تطوير حزمة الخدمات وتحسينها. وقد ساهمت الأنشطة التعاونية داخل المقاطعة بين مرافق توفير الرعاية الصحية إلى جانب تعاون العديد من القطاعات في توضيح اتجاه المستوى العام، والذي أدى بدوره إلى زيادة التركيز على البنية التحتية لخدمات توفير المياه والصرف الصحي ودعم ممارسات النظافة والعادات الصحية لتحسين النوعية الإجمالية لطريقة تقديم الخدمات.

**الدروس المستفادة:**

كانت القيادة على المستوى الوطني مهمة لتحديد الرؤية وتكوين بيئة مواتية لتغيير مفهوم خدمات المياه والصرف الصحي والعادات الصحية في مجال توفير الرعاية الصحية. وكان إشراك العاملين في مجال الصحة عاملاً مركزيًا في معالجة أمور البنية التحتية الأساسية وممارسات النظافة والعادات الصحية في مرافق توفير الرعاية الصحية وعملوا أيضًا كمحفزات للتغيير المستدام. وعلاوة على ذلك، من المهم إعداد خطة تنفيذ طويلة الأمد للمبادرات المقامة على المستوى الوطني لضمان الاستدامة.

## 摘要

**问题:**

医护机构缺乏适当的供水与卫生基础设施且卫生实践较差，这降低了其防范和应对疾病爆发的能力以及公众对所提供的医疗服务的信任。

**方法:**

为改善供水与卫生基础设施以及卫生实践，利比里亚卫生部举行了数次多利益相关方会议，以制定一项全国性供水、卫生设施、卫生及环境健康的一揽子计划。 并面向县级环境卫生技术人员开设了全国性培训师培训课程，包括感染预防、控制焦点人物以及充当变革推动者的焦点人物。

**当地状况:**

在利比里亚，参与调查的 701 家医护机构中仅有 45% 的医护机构在 2015 年对水源进行了改善，27% 的医护机构对感染性废物进行了适当的废物处理。

**相关变化:**

通过当地卫生工作者的参与引入当地所有权，以确保该一揽子计划的制定与完善。 医护机构之间的县级协作以及多部门协作强调了国家层面的指导，导致供水与卫生基础设施和卫生实践的实施引起了更多关注，以改善整体服务提供质量。

**经验教训:**

国家领导层能够确定未来方针并创造有利于改变人们对卫生保健供应中供水、卫生设施及卫生的认知的环境非常重要。 卫生工作者的参与是解决医护机构基本基础设施及卫生实践问题的核心，同时还充当可持续性变革的推动者。 此外，制定一项适于全国层面举措的长期实施计划对于确保可持续性非常重要。****

## Резюме

**Проблема:**

Отсутствие надлежащих объектов инфраструктуры водоснабжения и санитарии, а также плохие санитарно-гигиенические условия в медицинских учреждениях снижают подготовленность учреждений и их реагирование на вспышки заболеваний, а также снижается доверие населения к предоставляемому медицинскому обслуживанию.

**Подход:**

Для усовершенствования объектов инфраструктуры водоснабжения и санитарии, а также улучшения санитарно-гигиенических условий Министерство здравоохранения Либерии провело встречи с участием многих заинтересованных сторон с целью разработки комплекса национальных мероприятий по улучшению водоснабжения, санитарии и гигиены, а также по охране окружающей среды. Был организован национальный тренинг для инструкторов по охране окружающей среды, который включал профилактику инфекций и контроль над координаторами и в котором координаторы выступали в качестве инициаторов изменений.

**Местные условия:**

В Либерии только 45% из 701 обследованного медицинского учреждения улучшили состояние источников водоснабжения в 2015 году и 27% из этих медицинских учреждений имели надлежащую систему утилизации инфекционных отходов.

**Осуществленные перемены:**

Обеспечение разработки и усовершенствования комплекса мероприятий осуществлялось на местном уровне путем привлечения местных медицинских работников. Сотрудничество между медицинскими учреждениями в рамках округа наряду с межотраслевым сотрудничеством повлияло на руководство на национальном уровне, что позволило уделять повышенное внимание состоянию объектов инфраструктуры водоснабжения и санитарии и способствовало внедрению гигиенических практик для повышения общего качества медицинского обслуживания.

**Выводы:**

Руководство на национальном уровне имело важное значение для определения концепции и создания благоприятных условий для изменений в понимании организации водоснабжения, санитарии и гигиены при предоставлении медицинского обслуживания.****Привлечение работников здравоохранения имело решающее значение для решения проблем, связанных с основными инфраструктурными объектами и санитарно-гигиеническими условиями в медицинских учреждениях, а также являлось стимулом для устойчивого изменения. Кроме того, разработка долгосрочного плана реализации инициатив на национальном уровне имеет важное значение для обеспечения устойчивости.****

## Introduction

Water, sanitation and hygiene in health-care facilities are preconditions for providing health care of good quality. Despite this knowledge, a 2015 report revealed that 38% of the 66 101 health-care facilities assessed in low- and middle-income countries had no source of water.^1^

In Liberia, a low-income country in western Africa, only 45% of 701 surveyed health-care facilities had an improved water source in 2015, and only 27% of health-care facilities had proper disposal for infectious waste (Ministry of Health, Government of Liberia, unpublished data, 11 October 2016). The 2013–2016 Ebola virus disease outbreak in the country emphasized the importance of cleanliness, sanitation and regular hand washing. During the outbreak, the majority of patients were fearful of seeking care within health-care facilities due to risk of contracting the virus. The poor compliance with infection prevention and control measures and poor water and sanitation infrastructures and hygiene practices within the facilities further contributed to the fear. Between August and December 2014, outpatient visits were 61% lower and antenatal care visits 40% lower than the same timeframe in 2013.^2^ The shortage of personal protective equipment and soap and lack of compliance with basic infection prevention and control measures – such as limited screening of patients, poor isolation facilities and training of health-care workers – contributed to 372 health workers acquiring Ebola virus disease, of whom 184 died.

Here we describe the efforts made to improve the quality of health services in Liberia, focusing on the development and implementation of a package to improve water and sanitation infrastructures and hygiene practices during and after the epidemic.

## Local setting

A survey of 701 of the 727 health-care facilities in Liberia showed that the majority of them are public (62%), followed by private for-profit (31%) and private not-for-profit facilities (7%). Most of the facilities are clinics (88%), while health centres account for 7% and hospitals 5% (Ministry of Health, Government of Liberia, unpublished data, 11 October 2016).

During the epidemic, the Liberian health ministry introduced an infection prevention and control focal person into each of the 15 county’s health management team. In some resource-constrained counties, the focal person also serves as the environmental health officer working on water, sanitation and hygiene. The focal person oversees the delivery of routine infection prevention and control measures and water, sanitation and hygiene health-care interventions at health-care facilities, in collaboration with the facility-based infection prevention and control focal person. The focal person is also responsible for county outbreak preparedness and response efforts related to water, sanitation and hygiene and infection prevention and control.

## Approach

To inform the development of a package of interventions to improve and monitor water, sanitation and hygiene, the health ministry, with support from the World Health Organization (WHO), first conducted a situational assessment in 63 health-care facilities. The assessment revealed challenges in water treatment, testing of water quality and health-care waste management, including segregation, handling, treatment and final disposal of waste, a lack of ash and placenta pits and lack of protective fencing in waste management areas. In addition, environmental management was shown to be poor.^3^ Subsequently, the health ministry held several multistakeholder meetings to develop the national water, sanitation and hygiene and environmental health package. The package is described in detail elsewhere.^4^ Briefly, it is divided into so-called hardware and software components. Hardware components are aimed at improving overall water, sanitation and hygiene infrastructure in health-care facilities, such as construction and maintenance of water points, toilets, hand-washing equipment, burial pits for autoclaved waste and placenta pits for the disposal of placentas and other body tissues. Software components are processes, management and practices aimed at preventing health-care associated infections, e.g. improved hand hygiene practices and implementation of waste management protocols. The package also describes behavioural change communication strategies for health-care workers and the community.

In 2015, the health ministry, with support from WHO and the United Nations Children’s Fund launched the package, using a systematic and multisectoral approach, involving health and water, sanitation and hygiene sectors. The overall aim of the implementation was to have complete country coverage on components for water, sanitation and hygiene as well as measures of infection prevention and control. The implementation involved county health teams, since these teams are critical as a first line of defence against potential future outbreaks and overall health systems strengthening.

A national train-the-trainer course was held for focal persons, medical directors, community health department directors, community health social administrators, coordinators for water, sanitation and hygiene, coordinators for infection prevention and control, and environmental health technicians. The objective of the course was to teach the trainer to train health workers in their county and to support the roll-out of the package. The course involved four sessions each lasting four or five days, and a total of 94 people were trained between November 2015 and February 2016. The training curriculum was both theoretical and practical, including techniques, such as hand washing. The trainees visited a facility to conduct an assessment and used the results to identify areas for improvement and suggested actions. The course was conducted in close collaboration with infection prevention and control efforts, which provided focal persons with sufficient technical knowledge to improve and monitor water and sanitation infrastructures and hygiene practices, including safe management and treatment of health-care waste. Participants received a certificate upon completion of the course. To ensure trainers’ involvement in future district and health-care facility level training, a database of certified county trainers was created and made available to all water, sanitation and hygiene partners. To catalyse institutional change, the health ministry launched in-county collaborations between health-care facilities, so-called twinning exchange, which included workshops and facility visits.

The development of the package was informed by a global risk-based management tool^5^ for improving water, sanitation and hygiene to support further improvements in water and sanitation infrastructure and hygiene practices in health-care facilities. The tool identifies risks and provides corrective actions that should improve health-care facilities’ water and sanitation infrastructures and hygiene practices and infection prevention and control measures.

Further, the national minimum standards for infection prevention and control include water, sanitation and hygiene components. To ensure compliance with the minimum standards, the health ministry, along with WHO and relevant partners, conduct monthly facility assessments using the Liberia Health System Minimum Standards Tool.^6^

## Relevant changes

Key changes introduced in the health system included placing a strong emphasis on training of health workers at the facility level to deliver health services of good quality and to be prepared for future outbreaks. Local ownership, through engagement of local health workers, ensured development and refinement of the package. To improve effectiveness, accountability and efficiency of health programmes, multisectoral collaboration between environmental health; water, sanitation and hygiene; and health colleagues was put in place when developing the package. This exchange informed national level direction, which led to increased uptake of water sanitation and hygiene and infection prevention and control practices. Finally, the monthly facility assessments, which focused attention on functional infrastructures for water, sanitation and hygiene, were an important support for change. Also, the focus on accountability led to facility-based quality improvement processes to facilitate further improvements.

The changes have led to high engagement on improving water and sanitation infrastructures and hygiene practices from all health workers involved, including the package’s inclusion in programmes for infection prevention and control training. The engagement has also contributed to broader efforts to improve drinking-water quality and its management through development of national guidelines and a national quality strategy. Similarly, to address identified problems on infection prevention and control and water, sanitation and hygiene standards, the health ministry, in collaboration with partners, launched training for health workers known as Keep Safe – Keep Serving^7^ during the epidemic. From August 2015, safe and quality service training was held for almost 8500 health workers^8^. Construction of temporary screening and isolation infrastructures addressed hardware issues, and incorporating infection prevention and control supplies (e.g. personal protective equipment) into the routine supply list ensured sustainability.

It is important to highlight that during this time of the outbreak, very little was being measured due to increased attention on containing and stopping the outbreak in Liberia.

After the epidemic, the government wanted to ensure that improvements made during the outbreak were sustained; the government has identified health-service delivery of high quality as a key investment area,^9^ resulting in the establishment of a quality management unit at the health ministry. The quality management unit, in collaboration with the department of environmental and occupational health, is responsible for water, sanitation and hygiene. The health ministry has presented a strategic vision for embedding quality improvement approaches into routine health service delivery at all levels of the health system. The vision emphasizes the importance of water, sanitation and hygiene and engagement from health workers, the community and partners.

## Lessons learnt

Challenges encountered during the implementation of the package included poor water and sanitation infrastructures and poor infection prevention and control measures, specifically hand hygiene practices, at health-care facilities. The lack of a long-term roll-out plan, despite national training-of-trainers undertaken, has resulted in incomplete national implementation of the package. Nonetheless, package components are being implemented in a wider effort to improve the quality of health services. The findings from further implementation of the package, combined with service delivery tools’ implementation, will inform future national direction for quality planning, control and improvement of health services.

The main lessons learnt are summarized in Box 1. National leadership and local ownership were the drivers of the improvements in water and sanitation infrastructures and hygiene practices and these improvements helped to re-establish trust between health-care providers and communities.

Box 1 **Summary of main lessons learnt**

- National leadership was important to identify a vision and to create an enabling environment for changing the perception of water, sanitation and hygiene in health-care provision.
- The involvement of health workers was central to address basic infrastructure and hygiene practices in health-care facilities.
- Improvements in water and sanitation infrastructures, hygiene and infection prevention and control practices helped re-establish trust between health-care providers and communities and increased the use of health-care services.

This paper provides some insights on how water, sanitation and hygiene can be embedded into health service provision. The efforts made during the epidemic to improve water, sanitation and hygiene were used as a catalyst by the government for improvement of the quality of health service provision. The progress includes improving infection prevention and control practices; increasing staff performance; improving outbreak preparedness; and enhancing community engagement. The strategies presented here could be adopted and adapted for low-resourced health settings facing similar challenges.
